# The path toward ectogenesis: looking beyond the technical challenges

**DOI:** 10.1186/s12910-021-00630-6

**Published:** 2021-05-13

**Authors:** Seppe Segers

**Affiliations:** Department of Philosophy and Moral Sciences, Bioethics Institute Ghent, Ghent University, Blandijnberg 2, 9000 Ghent, Belgium

**Keywords:** Ectogenesis, Bioengineered uterus, Artificial womb technology, Obstetrics, Neonatology

## Abstract

**Background:**

Breakthroughs in animal studies make the topic of human application of ectogenesis for medical and non-medical purposes more relevant than ever before. While current data do not yet demonstrate a reasonable expectation of clinical benefit soon, several groups are investigating the feasibility of artificial uteri for extracorporeal human gestation.

**Main text:**

This paper offers the first comprehensive and up to date discussion of the most important pros and cons of human ectogenesis in light of clinical application, along with an examination of crucial ethical (and legal) issues that continued research into, and the clinical translation of, ectogenesis gives rise to. The expected benefits include advancing prenatal medicine, improving neonatal intensive care, and providing a novel pathway towards biological parenthood. This comes with important future challenges. Prior to human application, important questions have to be considered concerning translational research, experimental use of human fetuses and appropriate safety testing. Key questions are identified regarding risks to ectogenesis’ subjects, and the physical impact on the pregnant person when transfer from the uterus to the artificial womb is required. Critical issues concerning proportionality have to be considered, also in terms of equity of access, relative to the envisaged application of ectogenesis. The advent of ectogenesis also comes with crucial issues surrounding abortion, extended fetal viability and moral status of the fetus.

**Conclusions:**

The development of human ectogenesis will have numerous implications for clinical practice. Prior to human testing, close consideration should be given to whether (and how) ectogenesis can be introduced as a continuation of existing neonatal care, with due attention to both safety risks to the fetus and pressures on pregnant persons to undergo experimental and/or invasive procedures. Equally important is the societal debate about the acceptable applications of ectogenesis and how access to these usages should be prioritized. It should be anticipated that clinical availability of ectogenesis, possibly first as a way to save extremely premature fetuses, may spark demand for non-medical purposes, like avoiding physical and social burdens of pregnancy.

## Background

Several research groups are investigating the feasibility of generating an artificial uterus [1–5]. A team from the University of Eindhoven recently received a €2.9 million grant to develop a prototype human artificial womb building on these findings [6]. A womb-like environment to support extracorporeal gestation is expected to advance pre-natal therapy, benefit neonatal intensive care, and potentially provide a novel solution for people who want to have a child but who are not capable of undergoing pregnancy [1, 5, 7–20]. These expected benefits are cited by medical professionals, scientists and ethicists alike and are often considered the driving forces towards a future clinical introduction of this technology [21, 22].

Arguments for the production of artificial uteri are also made under the heading of promoting reproductive freedom and equality among genders and sexual minorities [14, 15, 19, 21, 23–32]. Further, it is often suggested that artificial uteri could reconcile not ending the life of the fetus with the pregnant person’s autonomy to terminate pregnancy [15, 25, 33–38].

My aim is to balance these potential benefits against future challenges as recent scientific steps add to the urgency to explore these questions while the science is developing.

Artificial womb technology (AWT) is the common concept to describe the technological component of the process that is generally called ‘ectogenesis’: i.e. partial or complete gestation of a developing embryo or fetus outside the human body. Complete ectogenesis, on the one hand, means that gestation happens from conception to birth completely outside a human body. ‘Partial ectogenesis’, on the other hand, can be divided into two interpretations [39]. On one interpretation, ‘partial ectogenesis’ refers to the transfer of a partially developed embryo or fetus from the female body to an external womb for the remainder of the gestation period. On another interpretation, ‘partial ectogenesis’ refers to techniques already routinely practiced in neonatology through the use of incubators to sustain premature babies, as well as in reproductive medicine through, for instance, in vitro fertilization. On this latter reading partial ectogenesis has already become a limited reality [14, 34, 39, 40]. I mainly focus on the first usage, as this is the area where most of the discussion is currently situated. Moreover, as signaled by Romanis, the assumption that AWT is an extension of current methods in neonatal intensive care may overlook morally relevant differences between AWT and current practices [41, 42]. Similarly, Murphy has suggested to use the term ‘extra corporeal gestation’ (ExCG) instead of ‘ectogenesis’ or ‘AWT’ to distinguish it from the limited ectogenesis in case of current practices. In this article, a combination of these concepts will be used [40].

## Main text

### Ectogenesis: types and state of the art

AWT interest first appeared at the turn of the nineteenth century in the writings of Haldane (who coined the term ‘ectogenesis’). In the 1970s and 1980s this was followed by a feminist inspired revival focusing on the liberating versus oppressive features of reproductive technology. In the early 2000s, there was a reappearance with a certain optimism about how AWT could advance fetal surgery and provide a controlled milieu for fetal development [18, 43, 44].

The current, fourth wave of AWT interest, makes the topic more relevant than ever before. The renewed interest in ectogenesis today is exemplified by the recent breakthroughs in animal studies, of which the EXTEND design and the EVE platform are the most important cases [4, 5]. Both approaches successfully enabled development of lamb fetuses in a sterile environment, using extracorporeal perfusion [4, 5, 7, 18, 42, 44, 45]. These studies have shown significant improvement of AWT as it purportedly moves closer towards clinical application in humans, which is as yet an undeveloped technology [2, 4, 5, 13, 46, 47].

#### Donated uteri, bioengineered uteri, EXTEND protocol and EVE protocol

Ectogenesis can take different forms: ectogenesis and AWT do not completely overlap, and research into ectogenesis can, but does not necessarily involve tissue or bioengineering.

In 1988, Bulletti et al. reported the first in vitro culture of a human embryo for 52 h in a hysterectomized human uterus (obtained from patients with cervical carcinoma or leiomyomas) that was extracorporeally perfused with an oxygenated medium [48]. It would raise several ethical questions if donated uteri are used as an alternative to adoption or surrogacy in case of uterine factor infertility (as has recently been suggested by Bulletti and Simon) [9]. Such a scenario would minimally have to grapple with issues concerning informed consent for obtaining the uterus, concerns regarding instrumentalization of the donor, and questions about who can donate (only patients undergoing hysterectomy for medical reasons, or healthy donors as well?). The use of living donors in the context of uterus transplantations (UTx) has brought along similar concerns for the welfare of donors and respect for their autonomy, but statements have been made that it can be justified if valid and informed consent is given by the donor (after thorough counseling); if levels of harm are proportionate to the benefits and fall below an accepted threshold; and if attempts are made to minimize the use of living donors and any harms inflicted to them [49]. A variant of using donated uteri for ectogenesis, is so-called xenopregnancy where an embryo or fetus is placed inside a uterine carrier belonging to a different species [8]. This has been tried in non-human animals. As far as safety is concerned, there are at least risks of immune rejection of the fetus, inappropriate interactions between the fetal trophoblast and the endometrium of the carrier and cross-species transfer of infections [8].

In a different kind of venture, researchers are aiming to develop uteri through tissue-engineering [9, 10, 50, 51]. Organ tissue-engineering is aimed at creating organs analogous to native biological organs. This can be done, for instance, by creating 3D scaffolds using biomaterials or by means of 3D bioprinting [10]. Some advances have been made in attempts to produce organs like the bladder, lung, liver and vagina [10]. Knowledge to develop artificial uteri through tissue engineering, however, is still starting to take off and mainly focused on animal experimentation [10, 50]. Important challenges are the search for suited in vitro conditions and appropriate technology to regulate important parameters like perfusion pressure and oxygen [50]. The research that is currently being undertaken into the development of tissue engineered uteri is not presently aimed at enabling ectogenesis, however. The benefits of these studies, as mentioned by Hellström and colleagues, are in vitro research applications (such as endometrial cancer cell studies and drug screening applications), and possible future application in in vivo transplantation studies (with the goal of curing uterine factor infertility) [52]. Similarly, the ultimate aim of tissue engineered uteri cited by Campo et al. is the usability in reproductive medicine, not in the form of ectogenesis, but by way of transplanting the bioengineered uterus [10]. This does not rule out that researchers may in the future continue their efforts to develop bioengineered uteri for use in ectogenesis. Bulletti and Simon, for instance, have stated that results from this field of research “offer hope” to develop an artificial uterus that can be used not only for uterine transplantation, but also for ectogenesis [9]. In that event, crucial ethical work will be due on questions concerning safety and proportionality, liability for fetal harm in case of malfunctioning bioengineered uteri, justifiability of animal tests as part of preclinical development, and the acceptability of using (human) biomaterial as a source for the artificial scaffolds, including obtainment of informed consent if donated cells are used. Some ethical work has been done regarding bioengineering and bioprinting in general, but the specific perspective of bioengineering or bioprinting of uteri remains largely unexplored, especially when use in AWT is concerned [53, 54].

The most recent successes in ectogenesis, however, are being made by employing relatively simple sterile containers for the fetus, rather than using donated uteri or bioengineered uteri [4, 5, 16]. A distinction can be made between pump-driven systems and pumpless systems. Pump-driven (venovenous) systems use a pump to control drainage of blood to an oxygenerator. Pumpless (arteriovenous) models for oxygenation use the fetus' heart to pump the fetal blood from the umbilical arteries [1, 55]. Bird describes both systems as "feasible" and Metelo-Coimbra and Roncon-Albuquerque conclude that pump-assisted models "show many advantages" over pumpless models [1, 55]. In the past, preclinical animal models using pump-driven circuits for perfusion showed success in supporting the development of isolated fetuses, but this success seems limited when compared to the recent though well-documented feasibility of long survival with a pumpless extracorporeal system [5, 13, 55, 56].

The current advancements, then, are being made with pumpless designs. The state of the art suggests that substantial departures from the uterine physiology are not optimal for clinical application [2]. It has been reported that the ideal model for AWT should preferably mimic the circulation as it occurs in the intact fetal umbilical-placental unit, with perfusion determined by fetal cardiac output [2, 4, 5, 56]. In 2017, an application of the EXTEND protocol has resulted in the successful four week support of extremely premature fetal lambs, without apparent physiologic derangement or organ failure [4]. After making an incision in the uterus, the developing lambs were held in a closed ‘Biobag’ containing continuously-circulated synthetic amniotic fluid. In 2019, the EVE protocol yielded similar observations, demonstrating survival of healthy fetal lambs for five days, which was purportedly extended to 14 days in a subsequent set of unpublished experiments [5, 57]. More recently, the latter group kept more immature ewe fetuses for a period of nearly five days in an ex vivo uterine environment (also using a protective bath of artificial amniotic fluid), demonstrating normal somatic growth, normal cardiovascular performance and absence of infection and inflammation [5].

Besides improvements in extracorporeal circuit configuration and advances in oxygenator technology, significant progress has been achieved both in demonstrating the physiological effects of the artificial environment and the feasibility of extra-corporeal support of younger animals biologically comparable to a 22–24-week gestation human fetus [4, 5, 13, 16, 46, 55]. It is said that this will be the primary clinical target population of AWT as a life support platform for extremely preterm infants [4, 5] Yet, as hinted at by Sahoo and Gulla, it is likely that if found successful, its use may ultimately cover a wider period of gestation and possibly also facilitate a wider range of applications [47].

### Potential applications of ectogenesis

#### Ectogenesis as neonatal care: decreasing morbidity and mortality among extremely premature infants

While there is no evidence that AWT can presently support extremely preterm fetuses, the eventual translational target is to provide a milieu where the fetus can continue to develop in a uterine-like environment without physiological stress of preterm birth [1, 5]. Various researchers and commentators explicitly state that the aim of AWT research is to improve current standards of neonatal care by decreasing morbidity and mortality among extremely preterm infants who cannot survive with existing neonatal intensive care [1, 9, 13, 16]. Existing ventilation-based life support is believed to have reached an efficacy threshold, since the structural and functional immaturity of the lungs of critically preterm fetuses are incompatible with pulmonary gas exchange [4, 57]. To the degree that there is a duty to save or improve the prospects for preterms, and assuming that AWT will be convenient to this end, this provides reason to develop AWT, particularly if this technology is more effective than existing technologies [41]. Yet, as I will explore below, this expressed and (by some) hoped-for progress towards AWT comes with ethical concerns about social implications, possible undesirable outcomes in terms of offspring welfare, and about whether regulation can keep up with the pace of the current developments.

There is an ongoing debate about whether or not AWT could be framed as a continuation of already existing neonatal intensive care. Romanis, for instance, provides arguments to differentiate AWT from neonatal intensive care (which also relate to her position that subjects of ex utero gestation are unique human entities which she terms’gestatelings’), though authors like Kingma and Finn object that these arguments are insufficient [27, 41, 42, 58]. Yet, such a framing may have clinical implications, as it seems to suggest that it can be introduced as an innovative technology, not as experimental research [42, 59]. The experimental use of AWT on preterms will expose research subjects to unknown risks and will come with concerns about parents-to-be feeling pressured to consent to experimental procedures (see below) [42].

It is worth noting that the proof of concept for *partial ectogenesis* demonstrated by Partridge et al. and Usuda et al. is thus not intended as an *alternative to pregnancy* (viz. complete ectogenesis), but rather as an *improvement* of (and possibly replacement for) *neonatal care* [4, 5, 60, 61]. It can reasonably be held that partial ectogenesis brings a certain urgency with it as it will be possible long before complete ectogenesis [42, 60]. In addition, partial ectogenesis impacts the pregnant person, not in the least because of the physical translocation of the fetus to the artificial womb [60, 62, 63]. An incision is made in the uterus—resembling a Caesarean—to expose the fetus, after which it is transferred to the sterilized container [2, 4, 5]. It is likely that this intervention will be no less risky than a Caesarean section—with the potential to be significantly riskier. Caesareans are a form of major surgery and entail possible adverse consequences like risks of blood clotting and excessive bleeding, wound infection, and in cases of a previous Caesarean there is an expected increased risk of obstetric complications (e.g. heightened risks of hysterectomy, abnormal placentation, uterine rupture) [31, 62–65]. Kingma and Finn recently hypothesized that some of these risks are more likely after partial ectogenesis (as the uterine incision will be done at an early pregnancy stage, when the uterus is less stretched than in a term pregnancy, the scar may be comparatively bigger, thus increasing future risks of uterine rupture, abnormal placental implantation, etc.) [27]. This is also relevant for the expectation that ectogenesis will advance the field of fetal medicine, discussed in the next section.

#### Advancing fetal medicine and optimizing the fetal environment

Several authors suggest that fetal therapy (e.g. certain surgical operations) would be easier if it could be done on an extrauterine fetus [7, 14, 18, 43, 62, 66–68]. In utero fetal treatment entails risks like surgical complications for the pregnant person and a risk of rupturing the uterus [69, 70]. Some authors have noted, however, that even if (partial) ectogenesis could make ex utero fetal therapy possible, the transfer of the fetus to the external uterus will most likely still require a surgical intervention on the person’s body (unless full ectogenesis becomes possible) [62, 71]. Thus, concerns about the pregnant person’s physical wellbeing still remain. Segers et al. have noted that dominant guidelines in neonatal care often urge directive counselling to convince pregnant persons to undergo medical intervention for the benefit of viable fetuses [62]. Yet, if ectogenesis pushes the limit of viability (i.e. make more or even *all* fetuses viable) this might further reinforce pressures on pregnant persons to undergo fetal removal when fetal therapy is advocated [35, 39, 47, 62]. Usuda et al. nuanced this by stating that it seems unlikely that fetuses much below 20 weeks gestation could be maintained on an artificial placenta, as this would require catheterization of umbilical vasculature and possibly compromise the fetal heart [61].

There is a similar expected benefit of ectogenesis in terms of fetal wellbeing, not necessarily for facilitating fetal therapy, but for optimizing the fetal environment by closely monitoring nutrition, temperature, oxygenation etc. This could be welcomed as a way of providing a more secure uterine milieu for the fetus, but it may also put pressure on pregnant persons to undergo certain interventions for the benefit of the future child [14, 60, 62]. This may especially (but not only) be the case when the pregnant person’s behaviour is thought to be worrying, for example because of substance abuse [14, 60]. Some fear that a greater knowledge of the fetal development through ectogenesis could be used to justify greater control on normal pregnancies [32]. If ectogenesis is able not only to increase health outcomes for new-borns, but also to minimize maternal morbidity and mortality, natural pregnancy might be considered risky and the choice to gestate might become stigmatized, and maybe even blameworthy when negative health outcomes do result [23, 47, 72, 73]. Some authors worry that ectogenesis could therefore oppose the meaning that some people find in gestation and childbirth, exposing a conflict between wellbeing of the future child and autonomy of the pregnant person [23, 68, 72, 73]. There is reason to believe that especially for individuals from disadvantaged groups this may lead to increased pressure to use ectogenesis to secure the safety of the fetus, which, in turn, is a reason to be sceptical about the freedom-promoting potential of AWT (see below). As Cavaliere observes, there is also the additional risk that disadvantaged women may be viewed as “substandard gestators” compared to AWT, which might exacerbate and entrench existing social inequalities [23].

Finally, the possibility to scientifically control the fetal growth environment might, according to Tong and Murphy increase the striving for ideal fetuses in ideal milieus [44, 74]. A related topic that receives relatively little attention is how ectogenesis could make genome editing easier in fetuses, both for disease related and non-disease related traits [67]. As far as worries about non-therapeutic applications are concerned, it may be noted that most of these concerns are not new or unique to the ectogenesis case. This is not to deny that this covers some important questions about autonomy, equity, justice and disability discrimination [14, 75].

#### Enabling biological parenthood

A third, and according to Kendal “perhaps the strongest justification for promoting the development of ectogenesis” [58], is to offer a new solution to people who wish to procreate, but for whom pregnancy is impossible or particularly dangerous [8–10, 14, 15, 17, 21, 30]. This could help a variety of people, including individuals with a damaged (e.g. due to cancer treatment), diseased (e.g. due to congenital uterine malformations) or missing uterus (e.g. due to hysterectomy or uterine agenesis); transgender women; single men and gay male couples; pregnant people facing health complications who are no longer capable of carrying the fetus to term. In all cases, except the latter, this would require complete ectogenesis, which is highly speculative. Focusing on these applications therefore engenders expectations that may prove to be unrealistic.

The argument to invest in ectogenesis as a way to assist procreation in the above groups, would be largely based on the value of reproductive autonomy (involving the right to decide whether to have children, how, when, with whom, etc.). More generally in the context of assisted reproduction, it has been argued that the value of autonomy may indicate that precedence should be given to a person’s preferred way of becoming a parent, but that redirection towards alternative ways is appropriate if there are good reasons for such a redirection, such as safety, cost-effectiveness and equity considerations [76, 77]. Relatively little attention has been paid to this when it comes to ectogenesis as a means of providing a person with a child versus alternative means, like adoption, surrogacy and UTx. Interestingly, however, when ectogenesis is evaluated under this light, scholars mostly do this as part of an argument in favor of ectogenesis over these alternatives (and some authors (e.g. [26]) worry that this may further stigmatize alternative ways of family building, like adoption) [9, 19, 28, 30].

First, surrogacy is morally fraught and prohibited in several countries. Some of these complications have to do with the risk of coercion of the surrogate, liability if something happens to the fetus, and the placement of the child if intended parents and/or surrogate change their minds about raising the child [28, 78]. Second, UTx holds safety risks for the fetus, the recipient and the donor (in case of live donor surgery) [9, 10, 28, 78]. Other issues include the possible exacerbation of black market uterus trade, the lack of suitable donor organs and undue pressure on living donors [10, 78]. Positive attitudes towards UTx are largely motivated by the value ascribed to carrying a child in the womb—which at least for some seems to outweigh the associated risks—but at the same time society’s deep association between childbearing and femininity has been criticized as placing too high expectations on women to bear children [62, 78, 79]. Developing UTx to create childbearing opportunities might exacerbate these pressures. Adoption, finally, does not impose physical risks on the child, the intended parents or the donor, but it often is an onerous, costly and emotionally burdensome procedure [78]. Arguments can be found that adoption is preferable to ectogenesis, as the latter would not produce the social benefits that adoption would [80]. It is questionable, moreover, whether the unproven character and expensiveness of ectogenesis as ‘infertility’ treatment can be outweighed by the benefit of preserving the prospect of having a genetically related child [80].

In general, the pros and cons of each of these alternatives should be thoughtfully considered and weighed against those of ectogenesis.

#### Non-medical application: equality and freedom promoting potential

One of the most dominant ectogenesis related strands is the possible positive effect on women in terms of liberation from reproductive inequalities and the social inequalities built on the back of that [14, 15, 19, 21, 23, 24, 26–32, 74, 81–84] As analyzed by Cavaliere, this view consists of two separate, though closely connected arguments: the equality-promoting argument and the freedom-promoting argument [23].

Firstly, the equality-promoting argument largely concerns ectogenesis’ potential to increase equality between (i) men and women, and (ii) among women [23]. Ectogenesis could (i) redress physical, social and financial burdens associated with pregnancy and childbirth so as to produce more equality between the sexes, but also (ii) enable women who are currently unable to gestate children to have children in the same way as other women who are able to do so [23, 83]. Some scholars extend this argument to a broader scope of genders and sexual orientations, like gay men and transgenders [14, 26, 35, 85].

One issue here is whether such use of AWT is undue use of obstetric technology [81]. There is, however, no clear ethical reason why ectogenesis cannot be used to achieve a non-medical goal if that goal is valuable and acceptable [81, 86]. Yet, it is not evident that women’s exclusive role in the gestation of children is the origin of gender inequalities more generally [24, 72, 73, 81]. If not pregnancy but other biological gender differences, gender roles and/or oppressive social structures are the problem, ectogenesis will not resolve gender inequality. According to some authors, arguments to use AWT for such ends would better be framed as political provocations, in the sense that ectogenesis talk draws attention to current unequal distribution of burdens of gestation between genders [23, 85]. Other authors have even argued that rather than being emancipative, ectogenesis would instead reinforce male gendered dominance, allowing men to reproduce without women (at least in theory) and eventually even lead to "the end of the existence of females" [87]. It seems unlikely that AWT would lead to the end of either sex, and it appears that such arguments should too be read as political provocations. Further, a crucial, if not the main point, that Cavaliere makes about these political provocations, is that to deliver on the promise to promote equality for all women, defenses of ectogenesis need to advance a broad political perspective that is not limited to criticism of gender inequality, but that also includes concerns about social inequalities within the sexes [23]. If the context within which ectogenesis is developed and provided is blind to inequalities that set higher bars for certain potential beneficiaries to access it (such as members of ethnic minorities, disabled and socioeconomically disadvantaged individuals etc.), ectogenesis might actually serve to reinforce social inequalities.

Secondly, the freedom-promoting argument refers, on the one hand, to ectogenesis’ liberative potential from burdens, restrictions and risks of pregnancy and childbirth. On the other hand, it echoes the reproductive autonomy argument to show that ectogenesis would enable people to pursue their preferred parenthood project [23]. With ExCG, pregnant people could avoid pregnancy (full ectogenesis) or end pregnancy (partial ectogenesis) in favor of ex utero gestation to sidestep pregnancy-related discomforts (e.g. morning sickness, bad moods, swollen limbs, migraines, pain of childbirth, depression) [17, 30, 83, 88]. Authors like Kendal and Murphy also cite social benefits like avoidance of pregnancy related career breaks and not having to alter smoking or drinking habits [74, 83].

If ExCG becomes clinically available, it is likely that there will be demand for such usage (which does not imply that ends related to e.g. career advancements should be taken as something that is desired without exception [23]). Yet, as noted by Romanis, access to obstetric technology is presently mainly medically controlled, which goes against offering ex utero gestation without medical cause [17, 60]. The high cost of AWT is also cited as an obstacle for using it as a way to avoid these so-called ‘minor’ discomforts [17, 61]. Kendal, on the other hand, defends state sponsored access to AWT for everyone and for all pregnancy-related discomforts, since the technology's being accessible only to those who are able to pay for it might make the gaps of socio-economic inequality deeper [83]. State-covered access to ectogenesis, however, would take away scarce resources for other competing purposes, which would be problematic if everyone who could benefit from the technology would be eligible for reimbursement, knowing that pregnancy always carries with it physical, social and economic burdens, and a risk of injury.

Importantly, prioritization decisions are not limited to resolving which particular treatments should (not) be funded. As noted above, access to reproductive technology is structured by power relationships that raise respective (reproductive) justice concerns about cultural barriers that disadvantaged groups may experience [23, 73]. For AWT to live up to its freedom-promoting potential, much will depend on the conditions under which the technology becomes available [23]. This comes with normative questions about who from the eligible patient population deserves priority, and what values are mobilized to account for such decisions.

In general, decisions about the accessibility of ExCG (for various groups of people and for diverse reasons) should be the subject of a democratic debate, based on sound argumentation. Authors who are skeptical about the freedom and equality promoting argument assert that such considerations should at least also take into account possible effects on the future child’s welfare, societal inequities and, in case of partial ectogenesis, the innate risks of preterm extraction of a fetus for ex utero gestation [17, 66, 73]. For these reasons, it is argued that ExCG would be less acceptable for social considerations and for lesser discomforts, than for e.g. cases of dangerous pregnancy or as a form of neonatal intensive care [17]. Taking account of the multitude of possible applications of AWT and setting consistent eligibility criteria with attention to possible roadblocks that people may face as potential users, are among the most important challenges for organizing fair access to this technology.

### Social implications: abortion, fetal viability, fetal termination

The above indicates that ectogenesis may have important implications for human reproduction and gestation that go beyond a strict medical focus [39]. As noted by Schoberer et al., the transgressing nature of ectogenesis and the way in which it diverts from pregnancy as we know it, might hamper the social acceptance of ectogenesis [89]. Relatedly, Simonstein and Mashiach-Eizenberg’s study of public perspectives on ectogenesis indicated that the use of AWT was found much more acceptable if it is aimed at survival of extremely preterm fetuses and enabling motherhood in case of absolute uterine factor infertility, than it is to discharge women from the burdens of pregnancy [90].

Public consultations teach us little about how we should evaluate developments like AWT, but they do offer important information about how the public values such advancements and how/whether this keeps pace with new scientific and ethical developments and insights, which can help organize a dialogue on those topics. The most recent empirical work on ectogenesis is the study by Di Stefano et al. who surveyed a sample of Australian obstetricians and neonatologists [36]. The authors reported ambivalence among professionals about the desirability of ectogenesis and mixed views on whether it should become common practice to save extremely premature fetuses. Professionals also expressed uncertainty as to whether ectogenesis should result in restrictions in access to abortion [36].

There is a currently ongoing debate about how ectogenesis might affect the acceptability and regulation of abortion. Some authors believe that ectogenesis could provide a more acceptable alternative to abortion by separating two currently inseparable events: the evacuation of the fetus from the womb and the death of the fetus [19, 29, 38, 91]. Singer and Wells famously stated that this could mean the end of abortion as we know it, for they consider the right to abortion as a right to be free of unwanted pregnancy, not as a right to the death of the fetus [19]. More recently Simkulet concurred that this alone is a reason for antiabortionists to pursue ectogenesis technology [38].

Against this several reasons can be given why ectogenesis might complicate rather than end the abortion issue [92]. First there is some indication that women, regardless of their personal views on abortion, may be unsympathetic to ectogenesis as a ‘solution’ to abortion [34]. Cannolds fieldwork suggests that there may be a gap between how theoretical positions have considered ectogenesis as a solution for the abortion debate, and how women actually see this [34]. A major objection to abortion for women who were anti-abortion was the assumption that a ‘good mother’ accepts responsibility for the care of her fetus/child, which does not match with having it brought to term in an artificial womb and putting it up for adoption afterwards. Many pro-choice women demonstrated a similar concept of maternal responsibility, reporting that ectogenesis and adoption would leave them with a lingering sense of obligation toward the future child, while abortion would be a form of motherhood prevention. In interpreting these results, caution is in order due to the small, unrepresentative nature of the sample, the risk of making anachronous extrapolations, and the difficulty of full appeal to empirical findings in assessing emerging technology. Still, Cannolds work may indicate that abortion is often sought as a means to prevent motherhood, and, more specifically, self- or socially imposed attributional parenthood [34, 93, 94].

Some have argued that the harms of attributional parenthood may constitute a moral justification for killing the fetus and others have argued that it is a reason for recognizing a right to the death of the fetus [37, 91, 95–97]. This argument has been subject to substantial criticism (ranging from objections that someone who already is a biological parent cannot have the right not to become a biological parent, to comments that it is unclear that the alleged harm of attributional parenthood is sufficiently great [98, 99]). I can only explore this issue superficially here, though one of the most solid objections is provided by Mathison and Davis (who offer an analogy with surrogacy, gamete donation, and adoption), stating that in analogous cases we do not typically think that such alleged harm entails particular further rights for a biological parent [100, 101]. Other arguments in favor of a right to the death of the fetus include the assumed right to genetic privacy and the assumed property right of the genetic parents over the embryo/fetus. As regards the former, there is unclarity about what it means to have a right to genetic privacy. The most plausible understanding is one in terms of protection from misuse of our genetic material, though that raises the question why ectogenesis paired adoption would imply misuse of one’s genetic material, and if so, whether that entails a right to the death of the fetus [98–101]. Also, the property rights argument—which rests on the controversial premise that the fetus is property of the genetic parents—faces difficulties, for even if it is granted that they do own the fetus, it does not ipso facto follow that the genetic parents can have it terminated [98–101]. I can merely outline some of these most central arguments to this discussion on the right to the death of the fetus [102, 103]. This debate is ongoing and the idea that pregnant people would be morally compelled to use AWT rather than seek termination of the fetus is far from settled [104].

This is complicated further by the fact that early stage abortion differs considerably from, and involves less risk than, carrying a fetus until it can surgically be transferred to an artificial uterus [80, 104, 105]. Cohen has suggested that the more invasive the transfer surgery, the stronger the claim not to be forced to undergo it, which is possibly backed by the right to refuse treatment [106]. Also, in countries where no legal personality is bestowed upon the fetus inside the patient’s womb, the stronger point can be made that the legal right to bodily autonomy and bodily integrity of the party possessing legal personhood—here: the pregnant person—should be respected and upheld, even if the surgery in question turns out to be minor (this is not a settled issue however, as illustrated by the pervasive disagreement on the justifiability of forced caesareans; see e.g. [107]) [108, 109]. Moreover, if fetal extraction to an artificial womb could eventually become physically equivalent to early stage abortion, autonomy and reproductive freedom of the pregnant individual would, however, still be impacted [104]. This goes beyond bodily autonomy, because a (reductive) focus on abortion as a means to end pregnancy, and the respective plea for ectogenesis paired adoption in lieu of abortion, may overlook the possible autonomy violation in denying people the choice not to have children [104].

Further, in case of full ectogenesis and when a fetus is developing in an ectogenetic incubator, it has been said that both parents will have an equal say on ending the fetus’ life (if the right of termination survives, that is), since the main difference between them—the gestational burden of the pregnant person—would be levelled out [22, 35, 106, 110, 111]. Important questions then include whether either parent could, on the basis of negative reproductive rights, choose to have an ectogenetic fetus terminated, whether one parent would need the consent of the other parent or the state to do so, and whether the action of ‘switching off the machine’ would be subject to abortion laws [110, 111].

Another issue concerns viability. Since ectogenesis might push the limit of viability lower, abortion could become impermissible under legislations that tie abortion rights to the standard of viability [35, 47, 105, 106, 110, 112]. ‘Viability’ is open to multiple interpretations [62, 112]. Yet, as marked by Abecassis, many judges interpret the term in a broad sense, acknowledging that viability largely depends on the existing technical advances [110]. It is speculated that AWT will render embryos and fetuses technically viable from conception, if this technology can successfully support their development in an ectogenetic incubator [105, 110]. Yet, as noted above, scientists temper this view arguing that it seems unlikely that fetuses much below 20 weeks could be maintained in an artificial womb [61].

Finally, it can be noted that in the context of ectogenesis several authors avoid taking a stand on the moral status of the fetus, even though there is a rather wide scope of literature available on the moral status of the fetus more generally and its implications for abortion [113–115]. An important exception is the correspondence between Colgrove and Romanis [41, 58, 116, 117]. Colgrove’s argument holds that subjects of partial ectogenesis “are a type of newborn” (maintaining that common definitions of ‘live birth’ actually seem to apply to these subjects), that they share the same moral status, and thus deserve the same moral treatment as newborns. The argument continues that subjects of complete ectogenesis cannot be identified as newborns (definitions of ‘live birth’ do not apply here), but that they do share the same moral status as newborns.^1^ While Colgrove concludes that whatever protections apply to newborns should be extended to ectogenetic subjects, there is ongoing discussion—and rightfully so—of appropriateness of definitions in this context (e.g. describing the expulsion of the fetus from the uterus to the artificial womb as ‘being born’ seems to disregard that emergence of that entity from the process of gestation is a crucial criterion of a definition of ‘live birth’ that is not completely met by such a fetal transfer) [41, 58, 116–118]. Moreover, as explicated by Romanis, assigning a moral status does not in itself entail how entities should be treated [58]. She writes: “once the status is assigned we must then make moral judgements about whether that status justifies certain treatment” [58]. For that reason, addressing moral status may not be such an urgent issue, or at least much less pressing than practical problems such as how to select research participants for innovative technology [58].

### Psychology: the impact of ectogenesis on child and parent

One of the oldest concerns about ectogenesis is the worry that it may be psychologically harmful, with most of the attention going to the possible impact on children born through ectogenesis. Nearly one hundred years ago, Vera Brittain rejected ectogenesis claiming that natural gestation is essential for children, regardless of whether or not it is essential for mothers [44, 119]. This worry was repeated by Singer and Wells and is still rather dominant [19]. The focus largely remains the child’s psychological and emotional development, though somewhat more attention has been paid lately to the psychological impact on the adult who would not have the sensory experience of pregnancy in the case of ectogenesis. For instance, some pregnant women develop, what Sander-Staudt calls, a relational psychology through talking or singing to their fetus as a result of a kick or a turn [120]. Sander-Staudt refers to stimulation of contractions and milk flow as a result of physical and emotional sensations (also see ref. 122). On these grounds, Sander-Staudt raised the question whether the use of AWT might lead to “an increased biological, emotional, and physical distance between mothers and children, and hence to society in general” [120].

There is some understanding that a pregnant person’s oxytocin level may significantly promote expression of maternal behavior after birth [121]. The physical sensation of gestating may also play a role in the development of this bond, which has been used as an argument in favor of UTx. This argument for UTx is used despite the absence of pelvic nerve connections [62, 78, 122]. There is also no accumulated knowledge about long-term psychological effects of UTx on parents and future children [123]. On the other hand, Golombok et al. indicated that absence of a gestational link between parents and their child does not seem to have a negative impact on the psychological well-being of parents [124]. Moreover, concerns about the dependency of a mother’s bond on physically gestating her child are disarmed by experiential knowledge about good parent–child relationships among step- and adoptive parents, and father-child relationships [31].

Yet, this seems to lose sight of the fact that these scenarios differ from ectogenesis, in the sense that here the child has been gestated by *someone*, while ectogenesis is unprecedented in that there is no or only a partial gestational relationship between the child and a person by whom it is gestated [111, 120]. Because children have always developed in utero, it is hard to predict whether and how this will affect the psychological and relational potential of the child developed in an AWT. Sander-Staudt warns that if those children miss out on stimuli that are preconditions for human affection and intimacy, this could have serious psychological implications [111, 120]. We do not know just how much a child’s psychology is impacted by being gestated in utero, but consideration must be given to how isolation from the uterine milieu—including the pregnant person’s voice, moods, heartbeat and touch—might adversely affect the child’s emotional and neuropsychological development [89, 105, 120]. At the same time, ectogenesis may actually offer more opportunities to interact with the fetus before birth, especially for the father.

Some authors believe that this suggests that research into ectogenesis should aim “as much as possible to simulate other features of organic gestation besides mere minimal physical components of nutrition and waste removal” [120]. This poses significant epistemological obstacles: the psychological effects of ectogenesis on a human fetus cannot entirely be obtained from data in the animal model [19, 89, 120]. In addition, laboratory observations in the human model would only seem to yield insightful results if the experiment is allowed to run until the child is born, and thus possibly exposing it to harm [125]. The extent of the risk of psychological harm is hard to determine, and at this stage of the research it seems to be impossible to assess whether any such harm could outweigh the benefits of ectogenesis. Future research will have to explore how this can be evaluated through responsible experimentation and longitudinal studies.

### Future perspectives: safety, animal experimentation, human testing, follow-up

Further dissemination of AWT-related research can reasonably be expected, but there are important hurdles that need to be overcome prior to clinical translation. In light of the recent achievements in the animal model, some commentators have prophesized that these technological and theoretical advances will soon make AWT in humans a clinical reality [126]. Yet, interestingly, the researchers of the EVE protocol have recently signaled that introduction to the clinic in the near future would be extremely premature [13, 61].

Safety is one of the most important reasons. Some critical technical and scientific issues are related to the inherent spasticity of umbilical vessels, the limited scope of placental functions that current models in AWT can perform, the determination and administration of appropriate nutrition and hormones, the monitoring of organ development (including lung and brain maturation), the functionality and placement of catheters in the fetus, the stability of the circuit flow (and the related issue of dosing paralytics to minimize fetal movement that limits circuit flow), and, importantly, the significant difference between ovine-based model systems and human models [2, 55].

This latter concern is one important reason not to be over-confident about the expected application of AWT in humans soon. As noted by Sahoo and Gulla, the differences in the development of the fetal lung and brain in ewes compared to humans may have led to favorable outcomes in the animal model, indicating that the same result might not be replicated in human fetuses [47]. The current animal data and the substantial physiological differences between the test animals and humans do not demonstrate a reasonable expectation of clinical benefit yet [42].

Testing on animals with physiologies more similar to humans has been suggested [42]. Yet, this is morally controversial, especially when non-human primates are used. Ethical work will have to be done to evaluate and balance the moral cost of such research procedures in terms of compromised animal welfare versus the moral benefits of ectogenesis research [127]. For instance, the ethical evaluation of animal testing for the development of partial ectogenesis to ameliorate morbidity and mortality among extreme preterms is likely to differ substantially from the ethical assessment of such procedures to develop full ectogenesis to avoid burdens of pregnancy. Moreover, authors like Schultz for instance, are skeptical that no matter how far the science is perfected in the animal model, a standard of ‘reasonable assurance’ of safety in humans cannot be met [29].

Safe clinical use of human ectogenesis will, then, require extensive research on human subjects as well, including embryos and fetuses, which is rife with controversy. To test partial ectogenesis, Alghrani and Brazier have suggested the use of human fetuses that are conceived and gestated (for a limited period of time) in utero, but where the pregnant person is about to go into premature labor, or when the pregnant person’s health and/or that of the fetus requires that delivery is initiated at a stage when conventional neonatal care offers little hope of survival for the baby [128]. The acceptability of such a research trial is largely underexamined. Central questions that have to be considered here are the validity of the future parents’ consent and whether such a trial can be regarded as therapeutic research at all [42, 128]. When, in that case, the parents-to-be consent to such a trial for the sake of the intended benefit of the child, one will have to consider whether this can be said to be in the future child’s interest, taking account of the possibility that viability may come at the cost of severe disability. The experimental use of AWT on preterms will not only expose research subjects to unknown short- and long-term risks, but it will also come with concerns about the validity of parental consent in such emotionally challenging and distressing circumstances [42, 128]. Alghrani and Brazier mention that it may be hard to obtain such consent, for instance because people may prefer to let the child “die in peace” when they are informed that their child is highly likely to die or be severely disabled in case (s)he survives the procedure [128]. Parents-to-be may also feel pressured to consent, so adequate and understandable information should be offered about the risks and benefits, the alternatives and the purpose of the research [42].^2^ In this context, awareness of the so-called therapeutic misconception should be addressed, because willingness to consent might be due to expectations that the experiment will offer the best health outcomes for the child (which is doubtful) [42]. It is unlikely that parents of premature children with good or even mediocre prospects of survival through traditional neonatal care would volunteer for such experiments, leaving only fetuses with dire prospects, which are least likely to result in healthy babies. Another possible source might be the use of fetuses when a decision has been taken to abort [128]. Important questions here are whether persons who want to end their pregnancy can authorize use of the fetus for ectogenesis research. There will be a significant risk of fetal death or injury, yet, as noted by Alghrani and Brazier, the purpose of such research is the fetus’s survival, and slim though the chance may be now, if it survives, one will have to confront the question whether in that case it can be killed [128]. A third option, which is especially relevant for testing complete ectogenesis, is the direct implantation of an embryo into an ectogenetic incubator where it is completely gestated. This would probably result from moving boundaries at both ends of the prenatal spectrum. That is, on one end, the possibility to extend human embryo culture and, on the other end, the growing ability to keep preterms alive. In combination with AWT, this might enable to span pregnancy completely ex vivo. Here, the use of spare IVF embryos might be thought of, though it should be noted that almost every country that allows embryo research upholds the so-called 14-day rule, which stipulates that embryos should not be grown in vitro for longer than 14 days after fertilization. Scientific developments like extended culture systems for in vitro embryos are now provoking suggestions to revise this rule, but the technical feasibility of extending the time span for keeping embryos in vitro does not in itself entail the normative conclusion that this would be acceptable [14, 30, 130–132]. Ethical discussion and democratic debate will be necessary to evaluate whether the instrumental use and longer culture of embryos are proportionate to the goals of ectogenesis (both full and partial).

Lastly, the issue of how much safety testing is required is a recurring discussion in reproductive contexts and it is not unique to the case of ectogenesis that possible risks to future offspring are weighed against other values [133]. Singer and Wells, for instance, stated that ectogenesis must not go forward until researchers are confident that the procedure will do “no, or at least, very little,” harm to future generations [19]. These are contentious notions, that should at least be actualized in the light of presently known risk factors. While it has been noted that AWT ‘does not have to be perfect’ because pregnancy is not perfect either, the involvement of clinical professionals does, however, come with additional role responsibilities [84, 134]. It should also be noted that concerns about the offspring’s wellbeing are complicated by philosophical arguments that it is only morally wrong to bring a child into existence by means of a risky technology if that child’s life will be so bad that it would be better for the child not to live. Yet, this argument does not reflect a consensus position among philosophers and bioethicists as it sets the bar for reproductive risk taking at a very low level [135]. The future child’s wellbeing is regarded as an important moral concern, also if it is above the ‘wrongful life’ standard, implying that the ‘good’ that is supposedly done by bringing someone into existence does not necessarily trump the harm that is inherently linked to that same act. Where the bar of the future child’s welfare should be set is contested, though several guidelines exist. The ‘reasonable welfare threshold’, for instance, holds that medical professionals should not offer technology if presently known risk factors foreshadow a high risk that the future child would have a seriously diminished quality of life [136].^3^ This requirement, including the stipulation that physicians carry joint responsibility for the welfare of the child due to their “causal and intentional contribution to the parental project”, is said to apply to all medical interventions enabling procreation, which reasonably also includes AWT [136]. It is an open question if/when the use of AWT could meet this reasonable welfare principle. In the meantime, more debate on the threshold for acceptable risk in this context is desirable—with an active role for multiple stakeholders, including ethicists, professional societies and patient organizations [134].

For now, it is important to observe that scientists and ethicists have stressed that there is as yet no clinical evidence supporting experimental AWT in humans [42, 61]. Caution is due and eventual clinical introduction should happen in a limited and closely monitored trial, with follow-up starting during pregnancy and continuing after birth, yet also here issues of privacy and informed consent will have to be taken into account. AWT in humans is as yet not to be welcomed as an innovative treatment (let alone as an established technology), but rather as an experimental procedure [59]. Uncertainty should be further reduced through preclinical studies using animals and, if this is found to be proportionate, it should be studied whether and how involvement of human embryos can be managed during this research stage [134]. When all conditions of experimental research can be fulfilled, strict indications can be defined for managing AWT as an innovative procedure, with specific restrictions aimed at reducing avoidable risks in the application of the relevant technology. Here the testing of AWT as an alternative to conventional methods of neonatal intensive care might be a possibility [59, 134]. Long-term follow-up will have to be central in this innovation strategy, which will require the continuing consent first of the parents but then also of the maturing child [66, 137]. Especially for a technology like AWT where there may be complications in terms of organ development and regulation of crucial parameters like oxygen and nutrition, there should be a continuous follow-up with strict feedback when irregularities are noticed. Harmful outcomes on their physical as well as mental health may only become apparent after birth, and long-term follow-up will be important to minimize risks to future children born via AWT [66]. It is presently unclear how long participant data collection should be conducted post ectogenetic birth. Singer and Wells have suggested a follow-up period of six years, though this suggestion is not particularly substantiated and more elaborate reflections are required [125]. One may draw here upon similar discussions from the context of UTx, where an international registry has been announced to determine the long-term safety of the procedure. Apart from questions about the duration of follow-up—the United States Uterus Transplant Consortium recommends a minimum of 5 years of follow-up of medical, cognitive, developmental, behavioral and social parameters—crucial challenges with respect to autonomy, consent, privacy and welfare of participants are being discussed [129, 138]. Debates about how to manage trade-offs between the protection of personal information of participants and the interests of future beneficiaries of UTx may inform the future clinical introduction of AWT [129]. This also includes questions about how to obtain appropriate and ongoing consent, as well as concerns about difficulties with data anonymization due to the small pool of beneficiaries, which may also be the case for AWT due to the recommended gradual roll-out of the technology [129]. Further dissemination of AWT should only be allowed in case of established results that reassure safety and effectivity. An important consideration throughout these research phases, as well as when this technology becomes clinically available, is the question of liability for potential injuries during the gestation in the ectogenetic incubator [29, 47].

## Conclusions

AWT could improve neonatal intensive care, provide an alternative for surrogacy or uterus transplantation, advance pre-natal therapy, remedy gender inequity and enable termination of pregnancy without terminating the life of the fetus. Prompt clinical introduction, however, is unlikely given the many concerns for the welfare of children born through AWT. Crucial work remains to be done regarding whether and how experimentation on (which) human subjects could be organized without crossing an acceptable threshold of risk and with rigorous follow-up during pregnancy and continuing after birth, with due respect for privacy and informed consent. Experimental use of AWT will expose research subjects to unknown risks, both physical and psychological, and entail concerns about the possibility of consenting to such procedures.

In setting conditions of experimental research, clear indications will have to be defined to determine acceptable application of the technology. AWT will primarily be used as a life support platform for extremely preterm infants, though it is likely that its eventual use may ultimately cover a wider period of gestation and possibly facilitate a wider range of applications.

In case of partial ectogenesis, the transfer of the fetus to the ectogenetic incubator will impact the pregnant person, meaning that respective concerns about physical wellbeing and autonomy still remain. If AWT would ever be used to advance fetal therapy, this might complicate the possible conflict between the beneficence towards the future child and the autonomy of the pregnant person.

AWT as a new solution for people who wish to procreate, but for whom pregnancy is impossible or risky, is largely based on the value of reproductive autonomy. This lays out the importance of balancing the pros and cons of other parenthood alternatives against those of ectogenesis. Considerations in terms of safety, cost-effectiveness and equity of access will be key. Given the multitude of possible applications—including the possibility to sidestep lesser pregnancy discomforts and related social burdens—setting consistent eligibility criteria will be one of the most important challenges for organizing fair access to AWT. Finally, societal debate is due about the impact of ectogenesis on the acceptability and regulation of abortion. Contrary to common assertions, ectogenesis might complicate rather than settle the abortion issue.

## Data Availability

Data sharing is not applicable to this article as no datasets were generated or analyzed during the current study.

## Abbreviations

AWT: Artificial womb technology
ExCG: Extra corporeal gestation
UTx: Uterus transplantation

## Footnotes

1 To make this point Colgrove invokes a hypothetical scenario that is to show that there is no reason to assume that subjects of complete and partial ectogenesis are unequal in some morally relevant way.

2 Similar reflections about consent and accurate information about the current state of knowledge of risks and benefits have been made in the context of UTx. See [129].

3 This is the intermediate standard between the minimal ‘wrongful life’ standard and the ‘maximal welfare’ standard. The latter rejects all medical interventions with indications that the life conditions of the future child will not be optimal [136].
